# Hybrid *in vitro* diffusion cell for simultaneous evaluation of hair and skin decontamination: temporal distribution of chemical contaminants

**DOI:** 10.1038/s41598-018-35105-z

**Published:** 2018-11-15

**Authors:** Hazem Matar, Nevine Amer, Sneha Kansagra, Andreia Pinhal, Elliot Thomas, Scott Townend, Joanne Larner, Robert P. Chilcott

**Affiliations:** Research Centre for Topical Drug Delivery and Toxicology, Department of Pharmacy, University of Hertfordshire, Hatfield, UK

## Abstract

Most casualty or personnel decontamination studies have focused on removing contaminants from the skin. However, scalp hair and underlying skin are the most likely areas of contamination following airborne exposure to chemicals. The aim of this study was to investigate the interactions of contaminants with scalp hair and underlying skin using a hybrid *in vitro* diffusion cell model. The *in vitro* hybrid test system comprised “curtains” of human hair mounted onto sections of excised porcine skin within a modified diffusion cell. The results demonstrated that hair substantially reduced underlying scalp skin contamination and that hair may provide a limited decontamination effect by removing contaminants from the skin surface. This hybrid test system may have application in the development of improved chemical incident response processes through the evaluation of various hair and skin decontamination strategies.

## Introduction

The adverse health effects of exposure to hazardous materials can be mitigated by decontamination: the timely removal of contaminants that may be on or near to body surfaces^1^. The emergency services’ response to incidents involving large numbers of individuals is commonly termed “mass casualty decontamination”^2^; one such protocol is the “Ladder Pipe System” of decontamination (LPS), during which a pair of fire engines park in parallel to deliver a high-volume, low-pressure water mist into a corridor through which casualties pass (https://medicalcountermeasures.gov/barda/cbrn/prism/; https://www.nfpa.org/~/media/files/news-and-research/resources/external-links/first-responders/decontamination/ecbc_guide_masscasualtydecontam_0813.pdf?la=en)^1^. The vast majority of previous studies that evaluated the efficacy of various decontamination systems focused on removing contaminants from the skin surface^3–10^. In contrast, relatively few studies have investigated hair decontamination and there is currently no *in vitro* model to quantify the risk associated with spreading contamination from hair to the underlying scalp or lower body surfaces during wet decontamination processes (e.g. LPS). The limited number of previous hair decontamination studies utilised pig or human scalp skin^11–16^. Although anatomically correct, such models do not accurately reproduce the normal geometric coverage of hair over the underlying scalp skin. This is because hair naturally falls under the influence of gravity and so the number of hairs covering the scalp will tend to increase from the top of the head down. Therefore, a model incorporating a “curtain” of hair laid over a skin surface would seem more representative.

We have previously developed an *in vitro* diffusion cell model that reproduces LPS hydrodynamics, allows the skin to be placed in a more realistic (vertical) geometry during showering and has a relatively large (~20 cm^2^) area to investigate the spreading of contaminants over the skin surface^17^. A logical step to develop this model further is the inclusion of human hair. Here we describe a hybrid *in vitro* model comprising excised pig skin partially overlaid with a curtain of human hair. The time-resolved, compartmental distribution of four chemicals—a curcumin and methyl salicylate mixture (CMX), sodium fluoroacetate (SFA), potassium cyanide (KCN) and phorate (PHR)—was investigated using the modified *in vitro* system. CMX has previously been validated for use as a simulant for medium volatility chemical warfare agents such as sulphur mustard^18^. The other contaminants were selected as being representative of toxic industrial chemicals.

## Results

**Figure 1:**
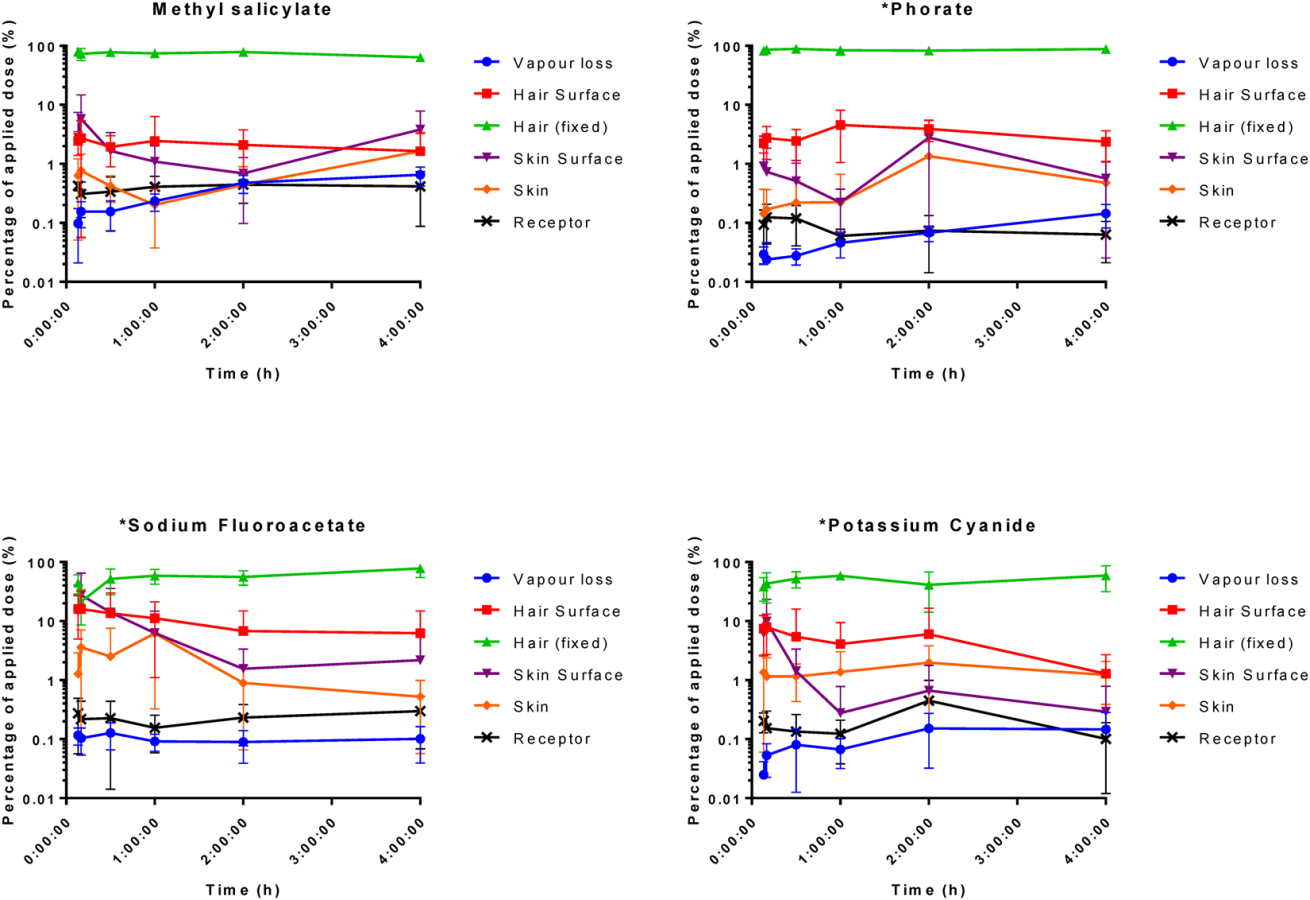
Distribution of ^14^C-radiolabelled contaminants—curcumin methyl salicylate mixture (CMX), phorate (PHR), sodium fluoroacetate (SFA) and potassium cyanide (KCN)—from different compartments (defined in Table 1) as a function of time post exposure. Each data point represents the mean recovery from n = 6 replicates, with error bars representing standard deviation.

A clear, consistent outcome was the predominant recovery of contaminants from within the hair (Fig. 1). When averaged across all time points, the greatest hair recovery was for PHR (85.8%; range 82.3–88.8), followed by CMX (75%; 63.9–79.0), SFA (51.2%; 21.3–77.8) and KCN (47%; 48.8%; 38.3–59.1). The hair recoveries of CMX and PHR did not differ statistically at any time (P > 0.05; two-way ANOVA, simple effects within rows, Tukey multiple comparisons post test). The same applied to the hair recovery of SFA and KCN. However, the hair recoveries of PHR and CMX were significantly greater than those of SFA and KCN at 8, 10, 30 and 240 minutes post exposure (P < 0.05). When contaminants were subcategorised as lipophilic (CMX and PHR) or hydrophilic (SFA and KCN), solubility and time were the most significant sources of variation (P < 0.0005; ordinary three-way ANOVA).

The second greatest proportion of applied dose was recovered from the hair surface (hair swabs): the time-averaged recoveries were SFA 11.5% (range 6.0–16.0), KCN 5.4% (1.3–7.9), PHR 3.0% (2.2–4.6) and CMX 2.2% (1.6–2.4), with SFA and CMX being statistically different (P = 0.0021; Friedman test with Dunn’s multiple comparisons post test). When subcategorised by lipophilicity or hydrophilicity, the hair surface recoveries were attributable to solubility (P < 0.0001) and time (P < 0.05; ordinary three-way ANOVA).

**Figure 2:**
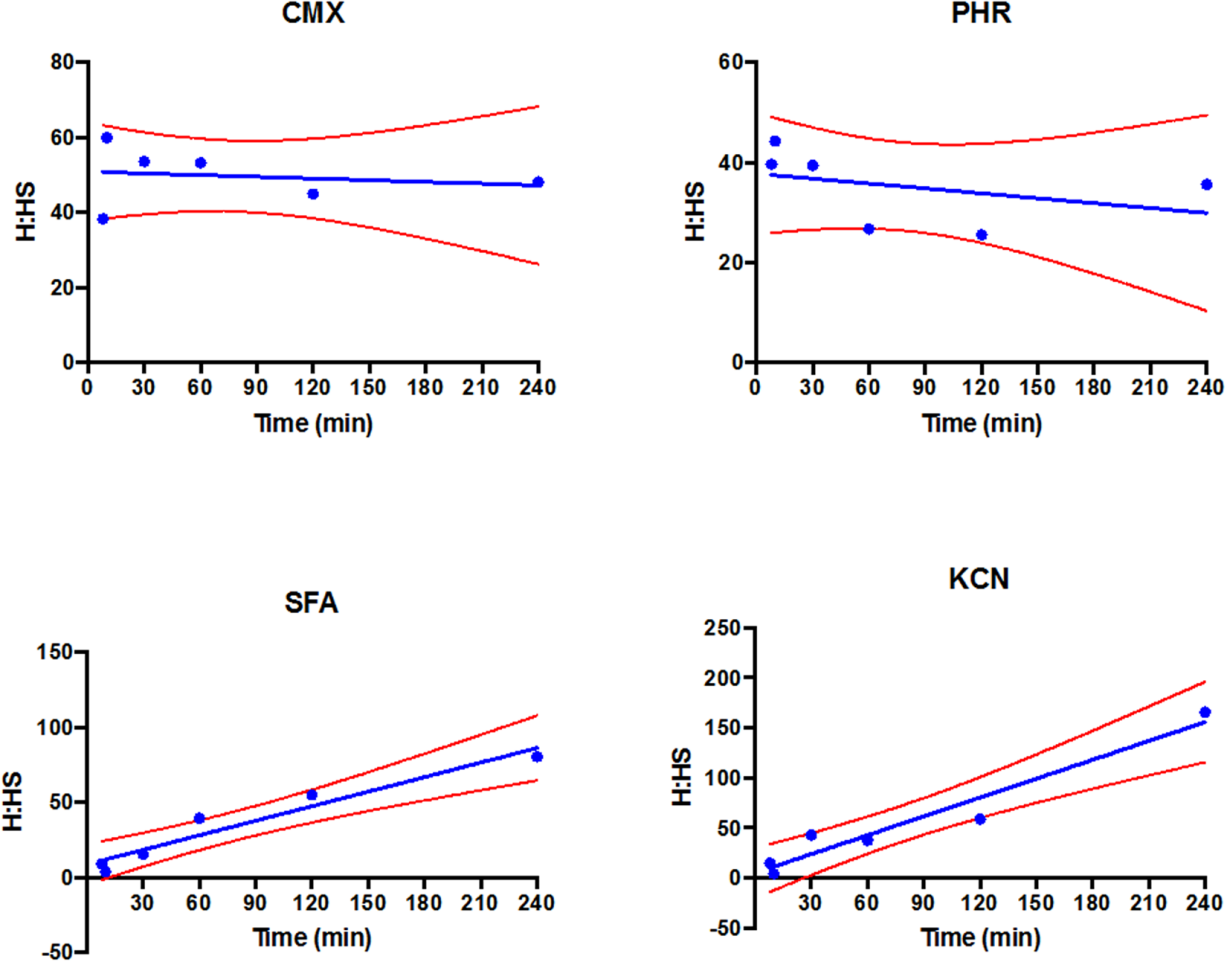
Distribution of ^14^C-radiolabelled contaminants—curcumin methyl salicylate mixture (CMX), phorate (PHR), sodium fluoroacetate (SFA) and potassium cyanide (KCN)—expressed as the ratio between recoveries from within the hair and the hair surface (H:HS) with time. Each point represents the average of n = 6 replicates. Red lines indicate the 95% confidence band of the line of best fit.

Given the statistically significant time-dependencies identified above, the hair-to-hair surface ratios (H:HS) of each contaminant were further investigated (Fig. 2). There was no statistical correlation for the H:HS ratio of CMX and PHR with time. However, there was a linear increase in the H:HS ratio for SFA (slope = 0.323 ± 0.043; P = 0.00217) and KCN (slope = 0.628 ± 0.07; P = 0.0014), indicating dynamic partitioning from the hair surface into the hair over the 4-hour exposure period.

**Figure 3:**
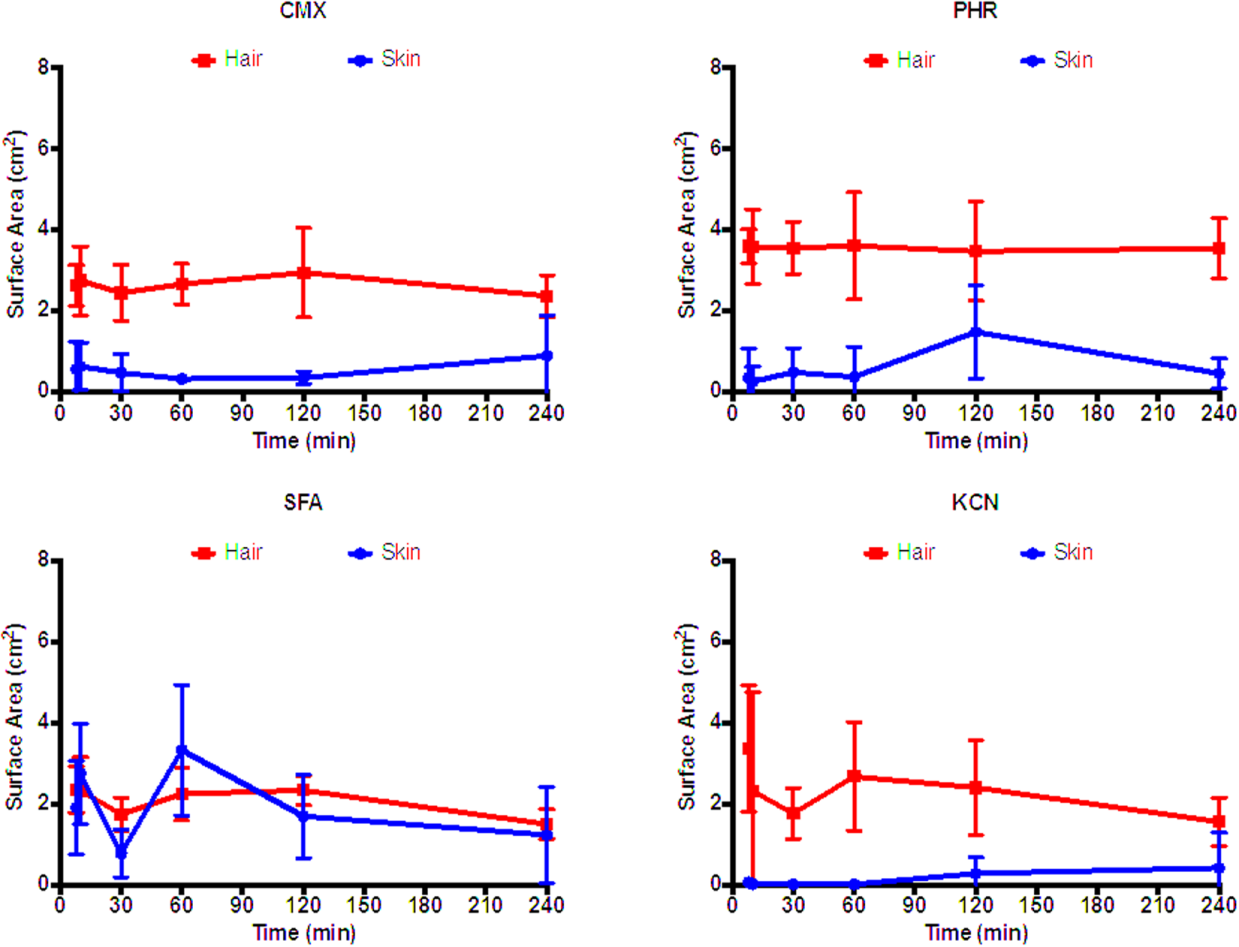
Surface area of hair and skin contaminated with ^14^C-radiolabelled curcumin methyl salicylate mixture (CMX), phorate (PHR), sodium fluoroacetate (SFA) or potassium cyanide (KCN). Each data point represents the mean of n = 6 replicates. Error bars indicate 95% confidence intervals.

**Figure 4:**
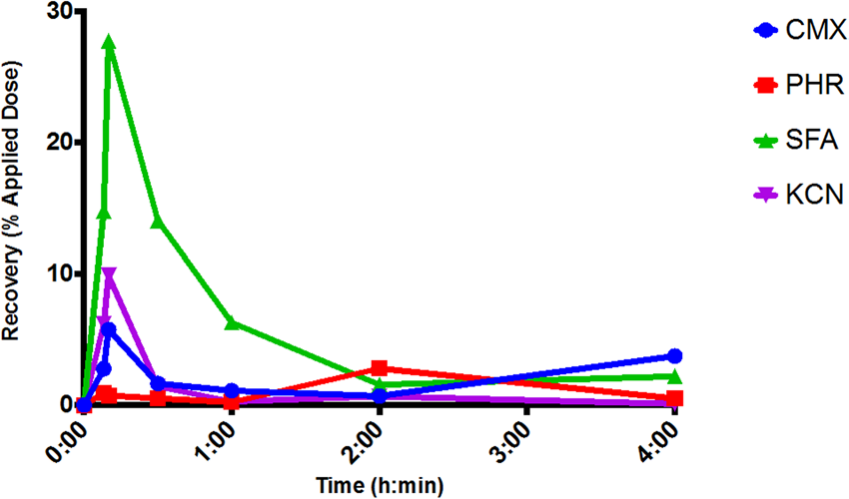
Recovery of ^14^C-radiolabelled contaminants—curcumin methyl salicylate mixture (CMX), phorate (PHR), sodium fluoroacetate (SFA) and potassium cyanide (KCN)—from the skin surface (skin swab) as a function of time post exposure. Each data point represents the mean recovery from n = 6 replicates. Error bars have been omitted for clarity.

The area contaminated with ^14^C-CMX, ^14^C-PHR and ^14^C-KCN was consistently and significantly (P < 0.05) greater on the hair curtains than on the underlying skin (Fig. 3). This was not the case for ^14^C-SFA, where there was no statistically significant difference between hair and skin (Fig. 3). There was also no significant correlation between either hair or skin surface contamination and time (P > 0.05), indicating that the spreading of each contaminant occurred within the first ten minutes of exposure (Fig. 4).

The initial recoveries of ^14^C-radiolabelled CMX, SFA and KCN from the skin surface appeared to reach a maximum at 10 minutes and then decreased thereafter (Fig. 4). This was not observed with PHR, but the inherent variability (coefficient of variance ~125%) precluded any statistically relevant comparisons between or within contaminants. The maximum skin surface recoveries were, SFA (27.8% of the applied dose) > KCN (9.9%) > CMX (5.8%). Since the amounts of contaminant recovered from within the skin and receptor chamber fluid were consistently lower than the skin surface recoveries (Fig. 1), the reduction of material recovered from the skin surface after 10 minutes could not be attributable to absorption into the underlying skin and receptor chamber fluid. There was a small but significant time-related increase in vapour loss of ^14^C-CMX (P < 0.0007) and ^14^C-PHR (P = 0.0002) with time (Fig. 1), which equated to evaporative loss rates of ~56 and 12 µg min^−1^, respectively.

**Figure 5:**
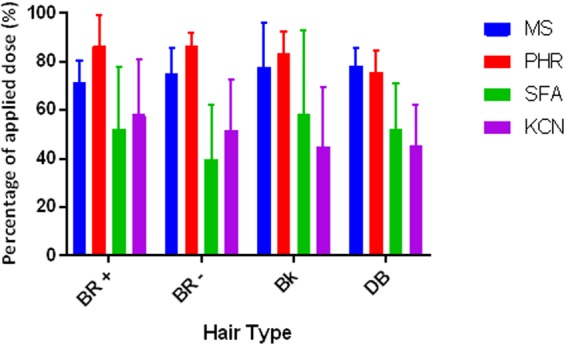
Percentage of applied dose of ^14^C-methyl salicylate (MS), phorate (PHR), sodium fluoroacetate (SFA) or potassium cyanide (KCN) recovered from different human hair types. A 20 µL droplet of ^14^C-MS, PHR, SFA or KCN was applied to human hair fixed onto dermatomed porcine skin. Each data point represents the mean recovery from n = 6 replicates, with error bars representing standard deviation.

There were no statistically significant differences between the different hair types employed in this study (Fig. 5). However, the recovery was consistently greater for hair contaminated with ^14^C-CMX and PHR compared to SFA and KCN.

**Figure 6:**
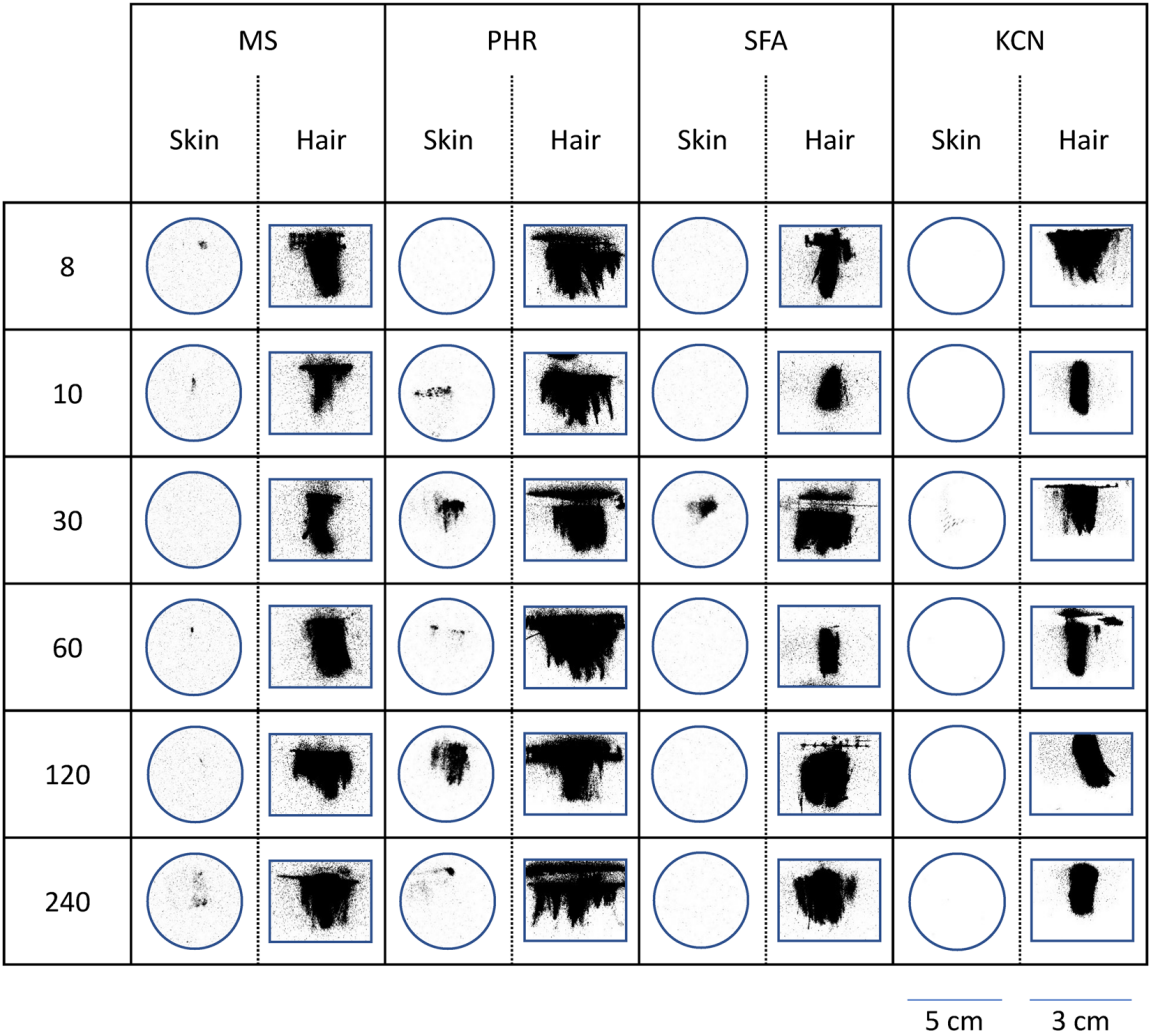
Representative autoradiograph images of hair and skin surface following exposure to ^14^C-methyl salicylate (MS), phorate (PHR), sodium fluoroacetate (SFA) or potassium cyanide (KCN). A 20 µL droplet of either ^14^C-MS, PHR, SFA or KCN was applied to human hair fixed onto dermatomed porcine skin to evaluate the effects with time. The extent of ^14^C-contaminant spreading was assessed 8, 10, 60, 120 and 240 minutes post exposure.

Lateral movement of each contaminant (across the hair or skin surfaces) did not significantly vary over the duration of the study (P > 0.05; one-way ANOVA with Dunn’s multiple comparisons post test), indicating that lateral diffusion was complete before the first time point (8 minutes). The spreading of each contaminant over the hair surfaces was consistently greater than the spreading over the underlying skin (P < 0.05; paired, two-tailed Wilcoxon test) with the exception of SFA (Fig. 6).

## Discussion

To our knowledge, this is the first description of an *in vitro* skin model that integrates a realistic “curtain” of human hair to investigate the time-dependent distribution of contaminants in different compartments (within the hair, skin surface, air sampling, etc.). Several methods have previously been reported for investigating skin decontamination using human scalp^12^, decontamination of human hair in the absence of skin^11,14^, and combined hair and skin decontamination using excised pig scalp skin^13^. Hair strands tend to fall under the influence of gravity to form a sheet over the underlying scalp skin. Therefore, a human hair curtain laid over dermatomed pig skin would appear to represent a rational approach for developing a more accurate hybrid hair/skin model.

**Figure 7:**
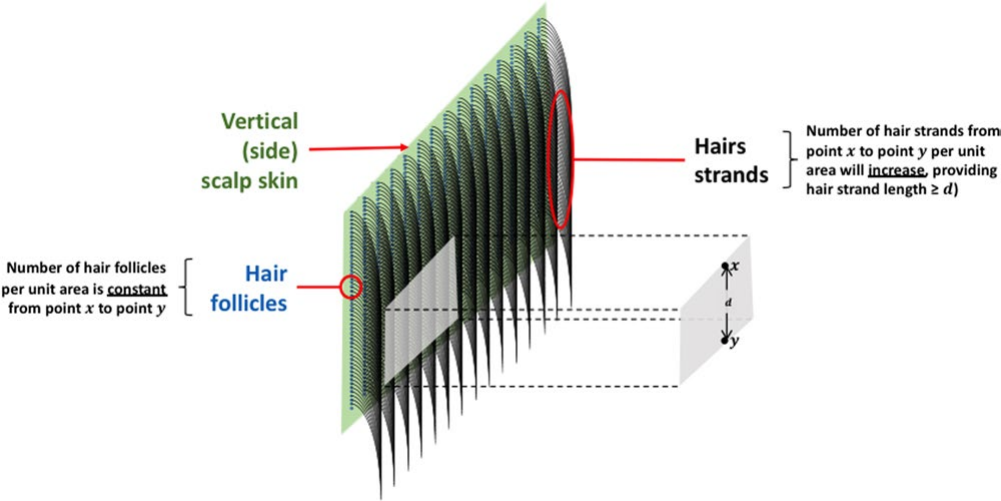
Schematic representation of hair geometry relative to underlying scalp skin.

The method for creating hair curtains was reproducible, with each swatch containing ~1785 hairs (±6%). The average number of hair follicles on a human scalp has been estimated at 350 cm^−2 ^^19^. Correspondingly, the linear arrangement of hair follicles would be √350 = 18.7 cm^−1^. The number of hair strands present over a section of scalp skin of similar dimensions to the hair curtain (3 × 1.7 cm) would thus be (3 × 18.7) × (1.7 × 18.7) = 1785. This calculation is based on the understanding that it is not the hair follicle density that dictates the amount of hair per unit area, but the accumulation of hair resting against the side of an individual’s head (Fig. 7). It is important to note that this *in vitro* model is only relevant for hair lengths in excess of 1.7 cm (represented by the distance *d* in Fig. 7). Thus, our hybrid model would not be applicable to individuals with shorter hair. The *in vitro* skin diffusion cell system used in this study has been described previously^17^ and is essentially a scaled-up version of a standard, validated diffusion cell system^20^. The relatively large surface area of our model provided adequate space for the introduction of the hair curtain. However, consideration should be given to the type of skin employed and the storage conditions of the skin (fresh or frozen) as this can affect dermal penetration^21^. This hybrid system uses previously frozen porcine skin, making this model slightly more conservative as frozen skin is more permeable to chemicals than fresh human skin^21^.

A surprising outcome of this study was the level of protection afforded by the hair, which retained the majority (53–89%) of the applied dose of each contaminant. In contrast, a previous *in vitro* study (using the nerve agent VX in a porcine scalp model) reported a hair recovery of ~10% of the applied dose, with the majority (75%) of the chemical distributed on or within the skin^13^. Since phorate and VX have similar physicochemical properties, this disparity may be explained by the different amounts and/or orientation of hair: the porcine scalp skin model mainly comprised vertical strands of hair at a density of 82 cm^−2^. In contrast, the human hair curtain in this current study was orientated horizontally and so provided greater coverage over the underlying skin. A recent human volunteer study reported a hair-to-skin ratio of ~20 for CMX at one hour post exposure^22^. This is of the same order of magnitude as the ratio of ~36 derived at the same time point for CMX in this current study. It should be noted that the volunteer study data would tend to underestimate the hair-to-skin ratio, as the hair was not excised and subjected to solvent extraction. Therefore, the *in vitro* model arguably provides a more realistic representation of the distribution of a contaminant between the hair and underlying skin. Collectively, these data indicate that scalp hair provides a significant degree of protection, a practical implication of which is that casualties exposed to toxic chemicals may be able to focus on other, less protected anatomical areas which may be more time-critical for effective decontamination.

No statistically significant differences were observed in the distribution of contaminants between the different hair types used in this study. Whilst subtle differences in hair types have previously been identified^23^, these do not appear to influence the distribution of contaminants following gross contamination.

An interesting observation was the dynamic redistribution of aqueous-based contaminants (SFA and KCN) between the hair and hair surface (H:HS ratio; Fig. 2). The latter was quantified as the recovery of chemicals from hair swabs, whereas the former was derived from solubilised hair following swabbing. Such compartmentalisation makes the assumption that swabbing is 100% efficient at removing unbound contaminant from the hair surfaces. It is conceivable that swabbing may underestimate the recovery of unbound contaminant to some degree (and thus artificially increase the H:HS ratio). Whilst care was taken to perform the swabbing in a reproducible manner, careful interpretation of these data is warranted. Taking this limitation into account, there was unequivocal evidence for the transfer of SFA and KCN from the hair surface to within the hair over the 4-hour exposure period. This effect was not observed with the lipophilic contaminants CMX (Log P = 2.23) and PHR (Log P = 3.67), the most plausible explanation being that partitioning of these lipophilic chemicals from the surface to within the hair was much more rapid and occurred before the first time point (i.e. within 8 minutes). This may be attributable to the presence of the sebaceous coating on the hair^24^.

A further interesting outcome of this study was the observation that recovery of contaminants from the skin surface increased transiently and then decreased 10 minutes post exposure (Fig. 4). This effect was most pronounced for the aqueous contaminants (SFA and KCN). One would expect the decrease in skin surface recoveries to be consistent with an increase in dermal absorption. However, the recoveries of contaminants from the solubilised skin and receptor chamber fluid compartments did not support this more obvious explanation. The fact that there was a corresponding increase in recoveries from the hair and hair surfaces after 10 minutes leads to the tentative conclusion that hair may absorb contaminants from the skin surface. A similar mechanism has been suggested to explain the re-coating of hair by sebum^24^.

A major limitation of this hybrid model is that it does not consider the impact of systemic absorption via the hair follicles, since the hair curtains were fixed to the skin surface and thus do not reproduce the normal anatomy of the scalp skin. Given that a (substantial) proportion of contaminants was recovered from within the hair, subsequent diffusion via the transappendageal pathways may be of toxicological relevance and so further work is required to assess the significance of this route.

## Conclusion

A hybrid model has been developed that combines dermatomed pig skin with a partial overlay of human hair. The hair layer provides a substantial degree of protection against exposure of the underlying skin, which is in agreement with a previous human volunteer study. Analysis of the temporal distribution of contaminants within the different experimental compartments has indicated a dynamic redistribution that is both time- and solubility-dependent. The two chemicals applied in aqueous solution (SFA and KCN) gradually partitioned into the hair over the 4-hour exposure period. In contrast, transfer from the hair surface to the hair was extremely rapid (<8 minutes) for the two lipophilic substances (CMX and phorate). There was evidence to suggest that hair may also have a limited capacity to remove contaminants from the skin surface. The hybrid system will be used in future studies to assess the effects of various dry and LPS decontamination strategies.

## Methods

### Materials

Methyl salicylate (MS; >99% purity) and curcumin (>98%, mixture of curcumin, desmethyoxycurcumin and bisdemethoxycurcumin) were purchased from Acros Organics, UK. PHR (95%) and SFA (99%) were custom synthesised by American Radiolabelled Chemicals (ARC, St Louis, USA). KCN (>98%) was purchased from Sigma Aldrich (St Louis, USA). Ethanol (Absolute), methanol (HPLC grade), propan-2-ol (HPLC grade), acetonitrile (HPLC grade), glacial acetic acid and formic acid (>99%) were obtained from Fisher Scientific, UK. Ultra-pure water (>18.2 MΩ) was filtered from the municipal supply via a MilliQ Integral 3 (Millipore, MA, USA). Compressed air, helium and nitrogen for thermal desorption gas chromatography were supplied by BOC UK Ltd. Nitrogen for the liquid chromatography mass spectrometer was supplied from a nitrogen generator manufactured by Peak Scientific Ltd., UK. Ring-labelled (^14^C) MS (70 mCi mMol^−1^), PHR (50 mCi mMol^−1^), SFA (50 mCi mMol^−1^) and KCN (58 mCi mMol^−1^) were purchased from ARC (St Louis, USA). Their non-radioactive analogues of MS and PHR were added in an appropriate proportion to give a final working solution with a nominal activity of 0.5 µCi µL^−1^ respectively. Curcumin was added to ^14^C-MS (10 mg mL^−1^) to produce the ^14^C-CMX solution. Saturated solutions of SFA and KCN were prepared by the addition of 2.5 mCi of each chemical to 5 mL water, with the subsequent addition of 5 g (SFA) or 3.5 g (KCN) unlabelled chemical.

Human hair (from 58 donors) was obtained from unisex hair salons within the Hertfordshire and Hampshire areas of the UK. Individual strands of hair were separated into four main types: dark brown (Br+), light brown (Br−), dyed blonde (DB) and black (Bk). Soluene-350 and Ultima Gold liquid scintillation counting (LSC) fluid were purchased from PerkinElmer, Cambridgeshire. Cotton wool was purchased from Robinson Healthcare, Nottinghamshire, UK.

### Hair and skin diffusion cell apparatus

**Figure 8:**
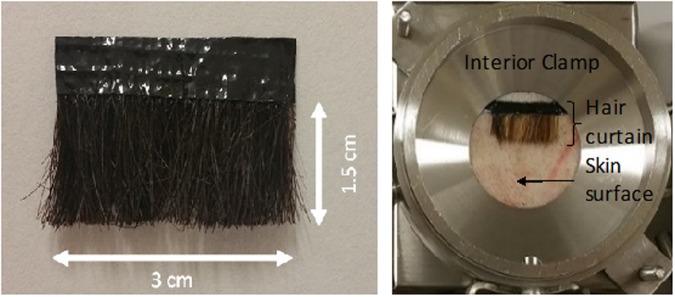
Representative example of a human hair curtain and the corresponding position on the surface of excised pig skin within a diffusion cell.

Each human hair curtain comprised ~1785 individual strands cut to a length of 17 mm. These were transferred to a strip of self-adhesive tape (3 × 0.4 cm). A thin film of cyanoacrylate glue was placed over the lower half of the tape to ensure that tip of each hair strand was embedded in the adhesive before the top of the tape was folded over the tip of the hairs. The resulting hair curtains (Fig. 8) were stored at room temperature for up to one month before use.

Skin diffusion cells and a manifold delivery system were manufactured by Protosheet Ltd. (Kent, UK) as previously described^17^. Full thickness skin was obtained *post mortem* from female pigs (*Sus scrofa*, large white strain, weight range 15–25 kg) from a reputable supplier. The skin was close clipped and removed from the dorsal aspect of each animal. The excised skin was then wrapped in aluminium foil and stored flat at −20 °C. Prior to the start of each experiment, a skin sample from one animal was removed from cold storage and thawed for approximately 24 hours. The skin was subsequently dermatomed to a thickness of 1000 μm (Humeca Model D80; Eurosurgical, Surrey, UK). Once dermatomed, the skin samples were cut into 10 cm diameter discs and mounted into the diffusion cells. Hair curtains were placed onto the skin 0.5 cm from the top (centre) of the interior clamp (Fig. 5) so that the side edges of the hair curtain were securely held in contact with the underlying skin. Each cell was connected to a peristaltic pump (Watson-Marlow 520S) through which receptor fluid (50% ethanol water) was infused at a rate of 0.5 mL min^−1^. Each diffusion cell was placed on a silicone heat mat connected to a digital controller (both supplied by Holroyd components, UK). The temperature of each heat mat was set to achieve a skin surface temperature of 32 °C (confirmed using an infrared camera; model FLIR P620). Diffusion cells were left for 1 hour to equilibrate, after which pre-weighed 20 mL glass scintillation vials were positioned at the receptor fluid effluent port to collect serial samples of receptor chamber fluid.

Experiments were performed in batches of six diffusion cells, with each allocated a specific treatment according to a pseudo-Latin square design (so that no treatment repeatedly occupied the same position within the fume cupboard). Each experiment was repeated six times to give a total of n = 6 replicates per treatment group, with each replicate being performed on skin from a separate skin donor (which was matched across all treatment groups). The hair type in each diffusion cell was randomised so that each experimental time point had at least one of each hair type. The treatment comprised exposure for a fixed duration (8, 10, 30, 60, 120 and 240 minutes) to one of the four test chemicals.

Each experiment was started by the addition of a 20 µL droplet of either ^14^C-MS, ^14^C-PHR, ^14^C-SFA or ^14^C-KCN directly to the centre of the hair curtain surface. Air from within each donor chamber was sampled using a constant volume pump (Pocket Pump model 210-1002MTX, SKC Ltd., Dorset, UK) set at a sampling volume of 75 mL min^−1^. Glass sorbent tubes were purchased from Markes International (Llantrisant, UK). Each tube glass tube was filled with 150 ± 5 mg of Tenax TA 35/60 absorbent. Filled Tenax tubes were conditioned using a TC-20 tube conditioner (Markes International Ltd., UK) in accordance with the manufacturer’s instructions.

After an appropriate delay (depending on the treatment group), the hair curtain was removed, placed into a pre-weighed 30 mL vial and stored at −70 °C for a maximum of 1 week. The skin and the inside surfaces of the diffusion cells were swabbed with dry cotton wool, which was placed into 10 mL ethanol; the diffusion cells were then disassembled and the skin samples were placed flat on a petri dish and stored at −70 °C for up to 1 week prior to autoradiographic analysis. The Tenax was removed from the glass sorbet tubes, placed into pre-weighed vials and then reweighed prior to the addition of 10 mL of propan-2-ol. The tubing connecting the donor chamber and the Tenax tubes were placed into pre-weighed vials containing 10 mL of propan-2-ol.

### Digital autoradiography

The skin and hair samples were removed from cold storage and placed into large (35 × 43 cm) autoradiography cassettes containing a radiometric calibration slide (30–862 nCi g^−1^; ARC, USA). A sheet of clear cellophane (38 μm thickness) was laid over the skin and hair samples and an erased phosphor imaging film (Fujifilm 20 × 40 BAS-MS, GE Healthcare, UK) was placed on top. Each film was exposed for 3 hours and then processed using a confocal, variable-mode laser scanner (Typhoon FLA 700, GE Healthcare, UK). Each scan was set to a resolution of 25 μm per pixel and a PMT of 800. Images obtained via autoradiography were spatially calibrated and analysed using ImageJ v1.48p to assess skin surface spreading using a (software) threshold of 244–62258. Regions of interest (ROI) were placed over the exposed area and each image was analysed. An average background signal (based on a ROI derived from negative controls) was subtracted from all analysed areas.

Once images were acquired, the skin samples and associated sections of cellophane were placed into pre-weighed 120 mL jars and reweighed before the addition of 50 mL of soluene-350 tissue solubiliser (PerkinElmer, UK). Hair curtains were blotted using a single sheet of absorbent paper (Wypall L20, Kimberly-Clark Professional, USA) cut into 5 × 5 cm swatches, which were then immersed in a 20 mL vial containing 15 mL propan-2-ol. Following blotting, each hair curtain and corresponding section of cellophane was placed into glass vials containing 20 mL of scintillation fluid.

### Sample analysis

Radioactivity of samples (swabs, Tenax, tubing, receptor chamber fluid, skin, etc.) was quantified using a PerkinElmer Tri-Carb liquid scintillation counter (Model 2810 TR), employing an analysis runtime of 2 minutes per sample and a pre-set quench curve. The amounts of radioactivity in each sample were converted to quantities of ^14^C-MS, ^14^C-PHR, ^14^C-SFA or ^14^C-KCN by comparison to standards (measured simultaneously) that were prepared on the day of each experiment by the addition of a known amount of test chemical (^14^C-MS, ^14^C-PHR, ^14^C-SFA or ^14^C-KCN) to (i) cotton wool swabs in 10 mL ethanol, (ii) Tenax and tubing in 10 mL propan-2-ol, (iii) individual hair curtains, and (iv) unexposed skin tissue dissolved in 50 mL soluene-350. A standard receptor fluid solution was also prepared by the addition of 10 μL of test chemical to 990 μL of fresh receptor fluid (50% aqueous ethanol), from which a range of triplicate samples (25, 50, 75 and 100 μL) were placed into vials containing 5 mL of LSC fluid to produce a standard (calibration) curve. Aliquots (250 μL) of each sample were taken and placed into vials containing 5 mL of liquid scintillation fluid for liquid scintillation counting. A summary of each sample type and its corresponding compartment is presented in Table 1.

**Table 1:** Summary of each sample compartment and associated description.

Compartment	Description
Vapour loss	Represents amount of contaminant that volatilised from the skin/hair surfaces, recovered from Tenax tubes.
Hair surface	Contaminant recovered from swabs of hair curtains.
Hair	Contaminant remaining in the solubilised hair curtains following swabbing. Assumed to be mainly representative of contaminant either trapped within the hair or bound to the hair surface.
Combined hair	Sum of recoveries from hair surface and hair.
Skin surface	Recovery of contaminant from cotton swabs of the skin surface and surrounding donor chamber.
Skin	Residual contaminant from within solubilised skin following skin swabbing.
Combined skin	Sum of recoveries from skin surface and skin.
Absorbed or receptor	The amount of contaminant recovered from the fluid bathing the underside of the skin.

### Statistical analysis

A normality test (Kolmogorov–Smirnov) was performed on all data. Equal proportions were not found to be normally distributed. Therefore, treatment effects were subsequently analysed by non-parametric tests using proprietary software (GraphPad PRISM v7.0a, USA).

## Data Availability

The datasets generated during and/or analysed during the current study are available from the corresponding author on reasonable request.
